# In Vitro Study of Antiamoebic Activity of Methanol Extracts of *Argemone mexicana* on Trophozoites of *Entamoeba histolytica* HM1-IMSS

**DOI:** 10.1155/2018/7453787

**Published:** 2018-07-30

**Authors:** Joel H. Elizondo-Luévano, Rocío Castro-Ríos, Eduardo Sánchez-García, Magda E. Hernández-García, Javier Vargas-Villarreal, Osvelia E. Rodríguez-Luis, Abelardo Chávez-Montes

**Affiliations:** ^1^Departamento de Química, Facultad de Ciencias Biológicas, Universidad Autónoma de Nuevo Léon, Ciudad Universitaria, 66455 San Nicolás de los Garza, NL, Mexico; ^2^Departamento de Química Analítica, Facultad de Medicina, Universidad Autónoma de Nuevo Léon, 64460 Monterrey, NL, Mexico; ^3^Centro de Investigación Biomédica del Noreste, Instituto Mexicano del Seguro Social (IMSS), 64720 Monterrey, NL, Mexico

## Abstract

Infections caused by parasites in humans represent one of the main public health concerns. Amoebiasis, a parasitic infection caused by *Entamoeba histolytica* (*E. histolytica*), is considered endemic in Mexico, where *Argemone mexicana* (*A. mexicana*) has been used in traditional medicine to treat intestinal parasitic diseases. The objective of this work was to evaluate the potential biological activity of *A. mexicana* on *E. histolytica*. For this purpose, a methanolic extract was prepared from *A. mexicana* leaves, and a differential fractionation was carried out with solvents of different polarities. The inhibitory capacities of the extract and its fractions were evaluated in vitro using HM1-IMSS, a strain of *Entamoeba histolytica*. *A. mexicana* extract was found to have a growth-inhibiting activity for *E. histolytica*, showing IC_50_ = 78.39 *μ*g/mL. The extract was characterized phytochemically, and the methanolic extract fractions were analyzed by liquid chromatography (HPLC) and mass spectrometry (MS). Berberine and jatrorrhizine were present in the active fractions, and these compounds may be responsible for the antiparasitic activity. The identification of amoebicidal activity of *A. mexicana* on *E. histolytica* gives support to the traditional use. Further studies with berberine and jatrorrhizine will be carried out to understand the mechanism involved.

## 1. Introduction

Parasitosis represents a global health problem, with intestinal parasites being one of the main causes of morbidity. This type of disease is closely linked to poverty and poor hygienic and sanitary conditions, so it appears more frequently in developing countries, especially in geographical areas where the ecological conditions favor the persistence of parasite [1]. *E. histolytica* is one of the most common parasites and is the causative agent of human amoebiasis. Infections with this parasite have different clinical manifestations, including diarrhea, dysentery, and liver abscess. Amoebiasis is acquired by ingesting *E. histolytica* cysts in contaminated food or water. This disease is a major health problem, according to the Global Burden of Disease Study conducted in 2013 [2]. Approximately 50 million people are affected by *E. histolytica* each year, and of these, approximately 100,000 die [3]. In fact, intestinal infections by protozoa, such as amoebiasis, are the third leading cause of death in the world.

The drug of first choice for treating amoebiasis is metronidazole, which interacts with the DNA of the protozoan [4], causing a loss of the helical structure and inhibiting the synthesis of nucleic acids, ultimately resulting in cell death [5–7]. The therapeutic use of natural products is as old as human civilization, and the vast majority of people on the planet continue to rely on the therapeutic properties of plants. In addition, the search for active ingredients from medicinal plants continues to provide active ingredients for treating diseases [2]. The exploitation of this potential source of medicines requires ethnobotanical, ethnopharmacological, chemical, biological, pharmacological, and toxicological studies [8].

*Argemone mexicana* L. (Papaveraceae), also known as Mexican poppy, has a wide distribution in many tropical and subtropical countries [9], and it is common to find it on the edges of roads, in vacant lots and on the sides of train tracks. Various types of chemical constituents are present in this plant, although alkaloids are the most abundant [10]. In traditional medicine, different parts of this plant are used to treat chronic skin diseases, ulcers, asthma, and other intestinal conditions [11–13]. Studies have shown that the extracts of *A. mexicana* and some of its individual components are effective against bacteria, fungi, viruses, nematodes, and parasites [14–18].

Considering the properties and background of this plant in relation to different microorganisms as well as the ease of obtaining the plant, the main objective of this study was to evaluate the activity of the methanolic extract of leaves of *A. mexicana* in vitro against *E. histolytica* HM1-IMSS and to identify the components present in the fractions of the extract because *E. Histolytica* represents one of the main etiological agents worldwide.

## 2. Materials and Methods

### 2.1. Vegetal Material

The leaves of *A. mexicana* were collected in the city of Monterrey, Nuevo León, Mexico, in February 2017. The material was identified as *A. mexicana* by the Department of Botany of the Faculty of Biological Sciences of the Autonomous University of Nuevo León. A voucher specimen was deposited with voucher no. 1208882 in the Herbarium of the Faculty.

### 2.2. Extraction

To obtain the methanolic extract of *A. mexicana*, the leaves were dried at room temperature and subjected to extraction by maceration [19]. For this, a 100 g portion was milled to a fine powder, and successive extractions (5 × 250 mL) were made with absolute methanol (Sigma Chemical Co., St. Louis, MO, USA) on an orbital shaker (Lab-line, 3508, Lab-line Instruments Inc., Melrose Park, IL, USA) at room temperature (24 h each). The obtained extract was filtered with Whatman no. 1 paper and concentrated under reduced pressure at 45°C using a rotary evaporator (Heidolph Rotary Evaporator, Laborota 4003, Heidolph Instruments GmbH & Co KG, Germany). Then, the residual solvent was evaporated at room temperature. The solid extract obtained was weighed to calculate the yield percentage and stored at 4°C until further use.

### 2.3. Fractionation of the Methanol Extract of *A. mexicana*

Serial partitions of the solid extract were made using solvents of different polarities and reactivity rates (Merck KGaA, Darmstadt, Germany). In the first stage, an extraction with hexane (3 × 100 L) was carried out, and the extracts were mixed and filtered with Whatman no. 1 paper (fraction A). The residue was extracted with 3 portions of 100 mL of CHCl_3_ (fraction B) and filtered again with Whatman paper, and the residual solid absolute MeOH (4 × 100 mL) was extracted and filtered with Whatman no. 1 (fraction C). Then, 100 mL of methanol was added to the solid and subjected to heating (120°C) for 5 min with stirring, followed by filtration (fraction D). Finally, the resulting solid was solubilized with water (fraction E).

### 2.4. The Activity of the Methanol Extract of *A. mexicana* against *E. histolytica*

The antiprotozoal activity of the extract was carried out with the microassay technique as previously described [7]. In brief, a concentrated solution of crude methanolic extract and another of berberine (1 mg/mL) in dimethyl sulfoxide (DMSO) at 5% v/v were prepared. This solution was sterilized by passing through a nylon membrane filter with a 0.22 *μ*m pore size (Merck Millipore, KGaA, Darmstadt, Germany) and stored, protected from light, at 4°C until use. Serial dilutions were performed by taking 500 *μ*L of the stock solution and adding 500 *μ*L of sterile deionized water. The concentrations of these working solutions were 0.031, 0.062, 0.125, 0.25, and 0.5 mg/mL. In culture tubes (Vial, Bellco Biotechnology, Bellco Glass Inc., Vineland, NJ, USA) with 1 mL of TYI-S33, 2 × 104 trophozoites of *E. histolytica* were deposited in the log phase, and 50 *μ*L of working solution was added to each tube. The tubes were incubated at 36.6°C for 72 h. Metronidazole at a concentration of 0.124 *μ*g/mL was used as a positive control, and 5% DMSO was used as a negative control. All bioassays were performed in triplicate and repeated three times [20]. After the incubation period, the tubes were cooled in ice water for 15 min, and the number of trophozoites per mL of each tube was counted with a hemocytometer (Neubauer, Hausser Scientific, Horsham, PA, USA).

### 2.5. Statistical Analysis

Growth inhibition percentages were estimated in reference to untreated controls. The extract concentration that would inhibit in 50% the *E. histolytica* growth, that is, IC_50_ with 95% confidence limits [21], was calculated by probit analysis (SPSS 24.0, SPSS Inc., Chicago, IL, USA). The log dose (concentration) response relationship was used in order to obtain the linear probit model. A chi-square goodness of fit test was used for assessing probit model adequacy.

### 2.6. Phytochemical Tests

To determine the functional groups of the compounds present in the methanolic extract and its active fractions, conventional chemical tests were carried out [19]. These tests included 2,4-dinitrophenylhydrazine (carbonyl group), anthrone (carbohydrates), Baljet (sesquiterpene lactones), sodium bicarbonate (carboxyl group), ferric chloride (tannins), Dragendorff (alkaloids), Liebermann–Burchard (sterols and triterpenes), potassium permanganate (double bonds), Shinoda (flavonoids), sodium hydroxide (coumarins), Bornträger (quinones), and sodium bicarbonate (saponins).

### 2.7. HPLC Analysis and Mass Spectrometry

Fractions C and D were analyzed by HPLC and MS. To do so, 15 mg of the dry fractions were obtained, dissolved in 2 mL of a mixture of methanol-acetonitrile (50 : 50), and filtered through a 0.2 *μ*m nylon membrane (Millipore). For the analysis, a Dionex Ultimate 3000 UHPLC System (Thermo Fisher, Dreieich, Germany) with a UV-Vis detector and coupled to an LCQ Fleet mass spectrometer (Thermo Scientific) equipped with an electrospray ionization source and ion trap analyzer were used. The HPLC analysis was performed using a Kinetex PFP column (50 × 2.1 m, Phenomenex, USA). As the mobile phase, a mixture of an aqueous solution of formic acid (1%) and methanol was used, starting with 30% methanol and increasing linearly to 100% over 10 min, returning to the initial conditions in the 11th minute and conditioning for 15 min before the next injection. The mobile phase flow was 200 *μ*L/min, the column was maintained at 50°C, and the injection volume used was 0.1 *μ*L. Nitrogen was used at a flow of 40 units as a nebulization gas (sheath gas). Ionization was performed in a positive mode. The electrospray capillary voltage used was 5 kV, the desolvation capillary voltage was 43 V, and the temperature was 275°C. The lens tube voltage was set at 75 V. Data acquisition was performed in full-scan mode at *m/z* 50 to 70 and with mass/mass experiments used for the most intense ions in the collision-induced dissociation (*CID*) mode, adjusting the normalized collision energy to obtain an adequate fragmentation with an isolation width of 1 *m/z*, an activation RF voltage (*activation Q*) of 0.25, and an activation time of 30 ms. All experiments were performed in triplicate.

### 2.8. Nuclear Magnetic Resonance Spectroscopy (NMR)

^1^H-NMR and ^13^C-NMR experiments were carried out using a Bruker Avance III HD 400 (400 MHz) spectrometer equipped with gradients and a 5 mm multinuclear probe (Bruker Corp., Billerica, MA, USA). For analysis, 10 mg of berberine standard, the dried methanolic raw extract, and fractions C and D were dissolved in methanol-d4 with 0.3% TMS as a zero reference. NMR spectra were analyzed using Topspin 3.0 software (Bruker Corp.)

## 3. Results and Discussion

The yield of methanol extracted by macerating (41.2 g) the dried leaf of *A. mexicana* was 9.95% p/p. As seen in Table 1, the extract was positive for unsaturation, sterols, triterpenes, quinones, tannins, saponins, carbohydrates, alkaloids, and flavonoids. These results coincide with those of previous studies, which have reported the presence of terpenoids, flavonoids, phenolics, long-chain aliphatic compounds, and a few aromatic compounds that are other constituents of this plant [8, 15]. However, it must be noted that the chemical composition of the plant may differ according to the parts of the plant that are used, the harvest season, and the geographical area.

Before evaluating the activity of the extract, it was necessary to assess the growth kinetics of *E. histolytica* to determine the logarithmic phase. Its generation time was found to be 6.3 h, and its doubling time was 4.3 h. After 72 h, the exponential phase begins, in which the parasite enters a period characterized by cellular duplication and is in its most active metabolic phase [22].

**Figure 1:**
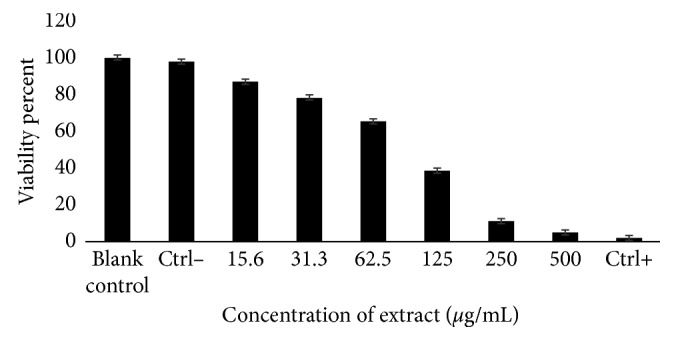
Evaluation of the viability of trophozoites of *E. histolytica*. Control: medium TYI-S33 inoculum of *E. histolytica*. Ctrl−: negative control (DMSO 5% inoculum of *E. histolytica*). Ctrl+: positive control (metronidazole 1 *μ*g/mL). The error bars represent the standard deviation of measurements in triplicate and repeated three times (*n*=9).

The activity assays against *E. histolytica* showed the ability of *A. mexicana* methanol extract to inhibit the trophozoite growth of *E. histolytica* in in vitro cultures under axenic conditions. As shown in Figure 1, the percentage of inhibition increases with higher extract concentrations. In addition, a negative control consisting of culture medium with 5% DMSO was evaluated for how it dissolved the extract inoculated with *E. histolytica*, and a positive control consisting of culture medium, inoculum, and metronidazole at 1 *μ*g/mL as well as a blank control containing the culture medium and inoculum was tested.

**Figure 2:**
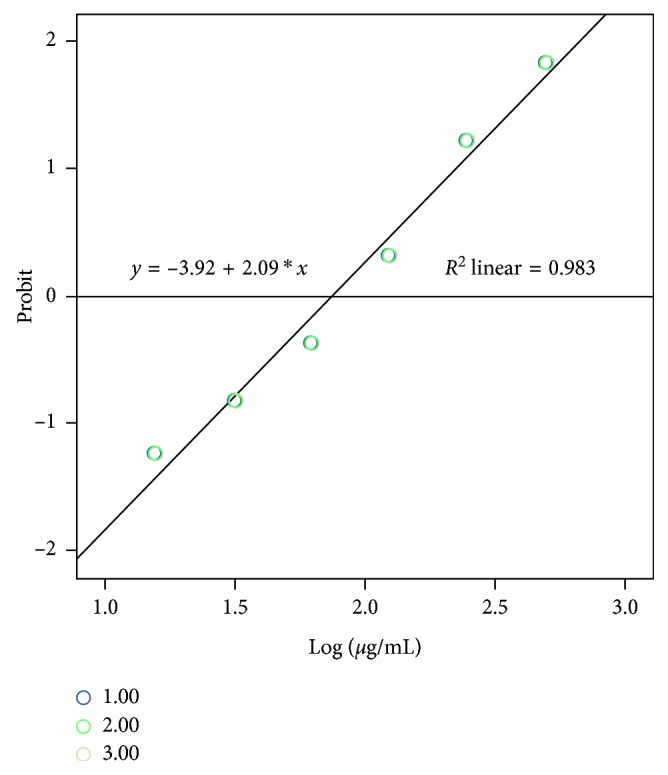
Probit graph of the activity of the methanol extract of *A. mexicana* against *E histolytica* HM1-IMSS. The IC_50_ of the methanol extract of *A. mexicana* on *E. histolytica* in their trophozoite form is shown and was performed in triplicate. IC_50_ for the methanol extract of *A. mexicana* on *E. histolytica* in their trophozoite form was 78.39 ± 0.48 *μ*g/mL.

The results showed a response behavior corresponding to the dose: as the concentration of the extract increased, the viability decreased. At a concentration of 500 *μ*g/mL, the inhibition was 96.6%. Even at the lowest test concentration of 15.6 *μ*g/mL, the extract still showed 8.8% inhibition. The negative control and the inoculum control had no activity against the parasite, but the positive control showed 99.6% inhibition. The determination of the mean inhibitory concentration against trophozoites of *E. histolytica* by the probit test showed that the methanolic extract had an IC_50_ of 78.39 ±0.48 *μ*g/mL (Figure 2), berberine of 40.65 ± 1.23 *µ*g/mL, and that metronidazole had an IC_50_ of 0.14 ± 0.02 *μ*g/mL. The result of the probit analysis applied to these data gave a chi-square value of 16,217 (*P*=0.300), which justifies that the data conform to the probit model, and the probit equations were obtained for each repetition: *P*=−3.929+2.09(*μ*g/mL), *P*=−3.935+2.09(*μ*g/mL), and *P*=−3.924+2.09(*μ*g/mL), highly significant results (*P*<0.01).

Although there are no reports on the use of extracts of *A. mexicana* against *E histolytica*, some studies have evaluated extracts of this plant against other parasites, such as nematodes [23]. The aqueous extract of leaves of *A. mexicana* exhibits significant anthelmintic activity against *Ascaridia galli* at a concentration of 100 *μ*g/mL, and this aqueous extract has also been used as an anthelmintic against the earthworm *Pheritima posthuma* [20]. Antiparasitic capacity against *Plasmodium falciparum* has also been demonstrated [9, 24], as the aqueous extract of the aerial parts of the plant exhibits activity against the chloroquine-resistant strain, with an IC_50_ value of 5.89 *μ*g/mL.

**Figure 3:**
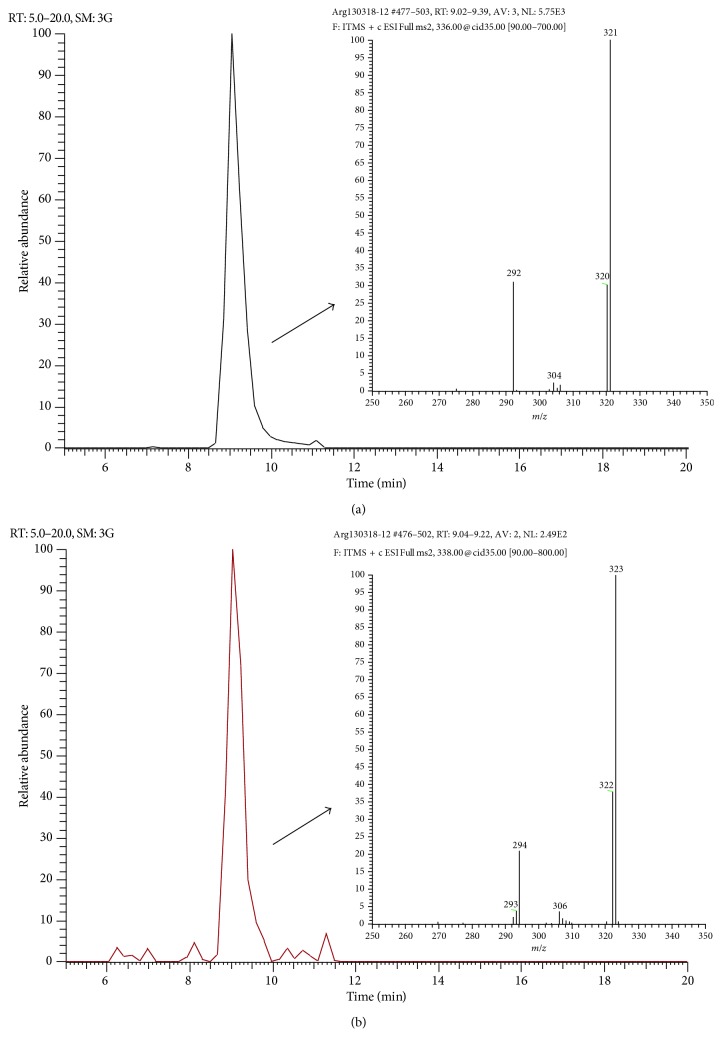
Chromatograms and mass spectra obtained for fraction D. LC-MS/MS fragmentogram and MS/MS spectra obtained for (a) berberine (*m/z* 336) and (b) jatrorrhizine (*m/z* 338) in fraction D.

**Figure 4:**
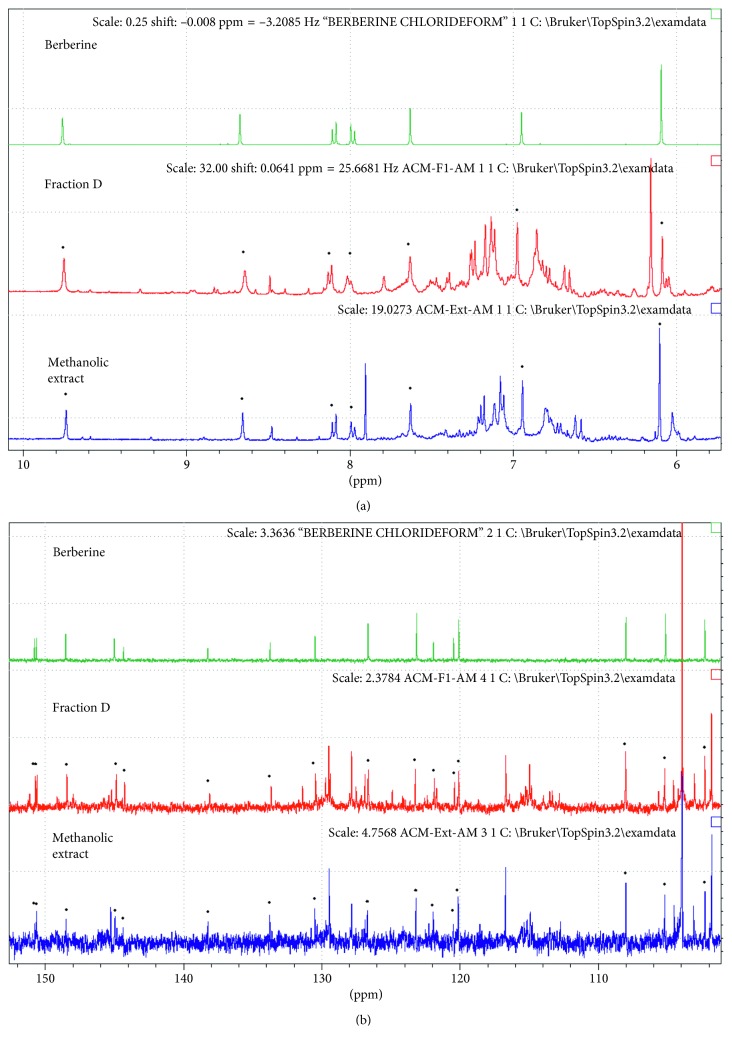
Comparison of (a) ^1^H-NMR and (b) ^13^C-NMR spectra of berberine, fraction D, and raw methanolic extract.

When the activity of the fractions of the methanolic extract was evaluated against the parasite, it was found that, at 125 *μ*g/mL, fractions C and D presented lethality percentages of 72.39 and 79.09%, respectively. Thus, these fractions were cataloged as active because the other fractions showed no relevant activity against *E. histolytica*. The active fractions (C and D) were analyzed by UHPLC-MS. Among the compounds that were present in both fractions, two important signals at *m/z* 336 and 338 were found and MS/MS experiments were carried out. Fragmentation of ion at *m/z* 336 yielded fragments at *m/z* 321, 320, and 292, while product ions obtained for ion at *m/z* 338 were 323, 322, and 294. The MS/MS spectra are similar to those reported for berberine and jatrorrhizine. As an example, Figure 3 shows the chromatograms and corresponding mass spectra for fraction D. In order to confirm the presence of the abovementioned alkaloids, ^1^H-RMN and ^13^C-RMN analysis were carried out for fractions C and D and the raw methanolic extract. For instance, in Figure 4, a comparison between the RMN spectra obtained for berberine, fraction D, and the raw methanolic extract is presented. As can be seen, for both the extract and fraction D, it is possible to observe the signals corresponding to berberine. The presence of jatrorrhizine could not be confirmed with these experiments, probably due to the low sensitivity of RMN, so further work on the isolation of the chromatographic peak that produces this signal will be made. These results agree with previous phytochemical investigations that revealed the presence of several alkaloids [11], including berberine, jatrorrhizine, protopine, allocryptopine, and sanguinarine. In addition, the plant produces more than 25 benzylisoquinoline alkaloids [25].

The compounds found in fractions C and D are of the alkaloid type and are probably responsible for the activity, as their effectiveness has been shown in extracts from plants of other genera that have been tested against the following parasites: *Giardia lamblia, Trichomonas vaginalis*, and *E. histolytica* [26]. Specifically, several pharmacological effects and activity against a variety of bacteria, fungi, protozoa, helminths, and viruses have been reported for berberine [27]. It has been demonstrated that berberine possesses inhibitory activity against *G. lamblia* and *T. vaginalis*, and in axenic cultures, morphological changes have been observed in the parasites by exposing these parasites to alkaloids, which caused a grouping of the chromatin in the nucleus and formation of autophagic vacuoles and aggregates of small vacuoles in the cytoplasm [28]. Also, using animal models such as the Syrian hamster, it has been shown that berberine has an effect against promastigotes of *Leishmania panamensis* and *Leishmania major* [29]. In addition, in a study conducted in 2014 [30], the antileishmanial activity of berberine against promastigotes of *L. major* and *Leishmania tropica* was evaluated. The results revealed that berberine was effective in inhibiting *L. major* and *L. tropica* promastigotes growth in a dose-dependent manner with IC_50_ values varying from 2.1 to 26.6 *μ*g/mL.

By contrast, jatrorrhizine, whose presence should be confirmed, is a protoberberinoid alkaloid and has been found in different plant species such as *Enantia chlorantha* and *A. mexicana* [31]. It has been reported to have an anti-inflammatory effect and may improve blood flow and mitotic activity in traumatized rat livers [32]. In addition, its antimicrobial and antifungal activities have beedn reported [17]. The activity of this alkaloid was also reported in vitro against *P. falciparum*, *Leishmania donovani*, and *Trypanosoma brucei rhodesiense* [32]. Therefore, the results obtained in this work validate the use of this plant as an antiamobic agent. This study also contributes to the search for new sources for the development of promising natural antiparasitic agents with possible applications in the pharmaceutical industry. More studies are being done to identify the synergy between the extract of the plant and standard antibiotics.

## 4. Conclusions

The methanolic extract of the leaves of *A. mexicana* showed growth inhibition activity against trophozoites of the *E. histolytica* strain HM1 : IMSS under axenic conditions in vitro. The analysis of the fractions of this extract by HPLC-MS showed greater amoebicidal activity, thus indicating the presence of berberine alkaloids and jatrorrhizine.

## Figures and Tables

**Table 1:** Chemical tests of the methanol extract of *Argemone mexicana*.

Phytochemical tests	
KMnO_4_ (double bonds)	+
2,4-dinitrophenylhydrazine (carbonyl group)	−
NaOH (coumarins)	−
Baljet (sesquiterpene lactones)	−
Bornträger (quinones)	+
Liebermann–Burchard (sterols and triterpenes)	+
NaHCO_3_ (carboxyl group)	−
Ferric chloride (tannins)	+
Saponins	+
Shinoda (flavonoids)	+
Anthrone (carbohydrates)	+
Dragendorff (alkaloids)	+

−: negative; +: positive.

## Data Availability

All the data supporting the findings of this study are available within the article. Nevertheless, interested researchers requiring further information can obtain them from the corresponding author upon reasonable request.
